# Using a Markov simulation model to assess the impact of changing trends in coronary heart disease incidence on requirements for coronary artery revascularization procedures in Western Australia

**DOI:** 10.1186/1471-2261-10-2

**Published:** 2010-01-06

**Authors:** Haider R Mannan, Matthew Knuiman, Michael Hobbs

**Affiliations:** 1 Department of Epidemiology & Preventive Medicine, School of Public Health & Preventive Medicine, Monash University, Melbourne, Victoria; School of Population Health, University of Western Australia, Perth, WA, Australia; 2 School of Population Health, University of Western Australia, Perth, WA, Australia

## Abstract

**Background:**

The population incidence of coronary heart disease (CHD) has been declining in Australia and many other countries. This decline has been due to reduced population levels of risk factors for CHD and improved medical care for those at higher risk of CHD. However, there are signs that there may be a slowing down or even reversal in the decline of CHD incidence due to the 'obesity epidemic' and other factors and this will have implications for the requirements for surgical treatments for those with CHD.

**Methods:**

Using a validated Markov simulation model applied to the population of Western Australia, different CHD incidence trend scenarios were developed to explore the effect of changing CHD incidence on requirements for coronary artery bypass graft (CABG) and percutaneous coronary interventions (PCI), together known as coronary artery revascularization procedures (CARPs).

**Results:**

The most dominant component of CHD incidence is the risk of CHD hospital admission for those with no history of CHD and if this risk leveled off and the trends in all other risks continued unchanged, then the projected numbers of CABGs and PCIs are only minimally changed. Further, the changes in the projected numbers remained small even when this risk was increased by 20 percent (although it is an unlikely scenario). However, when the other CHD incidence components that had also been declining, namely, the risk of CABG and that of CHD death for those with no history of CHD, were also projected to level off as these were declining in 1998-2000 and the risk of PCI for those with no history of CHD (which was already increasing) was projected to further increase by 5 percent, it had a substantial effect on the projected numbers of CARPs.

**Conclusion:**

There needs to be dramatic changes to several CHD incidence components before it has a substantial impact on the projected requirements for CARPs. Continued monitoring of CHD incidence and also the mix of initial presentation of CHD incidence is required in order to understand changes to future CARP requirements.

## Background

The population incidence of coronary heart disease (CHD) in Australia and many other countries has been declining for the last two to three decades [1]. In addition to age and gender, the incidence of CHD has been shown to vary with levels of key risk factors such as high blood pressure, high cholesterol, smoking and diabetes. In Australia, although the trends in major CHD risk factors such as blood pressure, smoking and cholesterol have been improving and have contributed to the declining CHD incidence, the prevalence of obesity and diabetes in the population are increasing [2]. This is evidenced by the fact that the prevalence of obesity in 1999-2000 was 2.5 times higher than the prevalence observed in 1980 [3]. Between 1985 and 1997, the prevalence of overweight and obesity doubled among young Australians with obesity alone trebling over the same period among young people [4]. In 1999-2000, 19% of Australian males and 22% of females aged 25 years or over were obese and an additional 48% of males and 30% of females were overweight based on measured height and weight data through the 2000 Ausdiab study [5]. The 2005 Ausdiab follow-up showed that since 2000 on average those aged under 65 at baseline had an increase in weight, BMI and weist circumference [6]. Obesity, especially abdominal obesity, increases blood pressure and total cholesterol levels [7-9], lowers high density level (HDL) cholesterol, and predisposes to type 2 diabetes [10]. Although studies of obesity as a direct risk factor for CHD have been inconsistent [11-15] and although there has been increasing use of medications to control CHD risk factors such as high blood pressure and high cholesterol [16], the obesity epidemic may soon result in a slowing down or even reversal off recent downward trends in the incidence of CHD [17,18].

Coronary artery bypass graft (CABG) and percutaneous coronary interventions (PCI), together known as coronary artery revascularization procedures (CARPs), are common and expensive treatments for people with coronary heart disease. The requirements for CARPs in a population are influenced by the incidence of CHD in the population and the effectiveness of CARPs and medical treatments. We have previously developed and validated a CHD/CARP Markov Simulation model for analyzing and forecasting the number of CARPs in the population of Western Australia [19,20]. We have also used this model to explore through scenario analysis the effect of recent advances in PCIs, namely, the introduction of drug eluting stents, on requirements of CARPs [21]. In this paper, we describe how this model can be used to explore the sensitivity of the requirements of CARPs due to the changing trend in CHD incidence as might be caused by the possible and likely effect of the obesity epidemic.

## Methods

**Figure 1:**
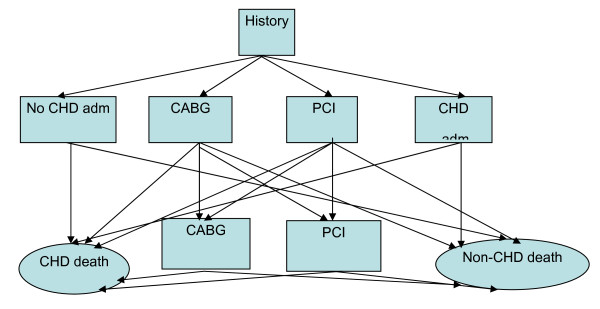
**Schematic diagram for the recognised events during a cycle (year)**.

The CHD/CARP Markov simulation model [22-24] is based on annual risks, stratified by age, gender and CHD/CARP history, for admission to hospital for CHD, for a PCI, for a CABG, for CHD death and non-CHD death. The model is applied to the cohort comprising the population of the state of Western Australia aged 35 to 79 years at the beginning of 2001. Using linked records for individuals in the WA Health Linked Data system [23] together with population census data, each member of the cohort is placed in one of several mutually exclusive CHD/CARP history states based on their hospital admissions history for the period 1980 to 2000: (1) a history of having a PCI but no CABG some time in the past; (2) a history of having a CABG some time in the past; (3) a history of having a CHD admission but no CABG or PCI some time in the past; or (4) no history of CHD admission, CABG and PCI in the past. The model allows members to move from one state to another each year according to the events they experience during the year. Within a year, the model allows for a CHD admission without a CARP, up to two admissions with CARPs (CABG or PCI) in addition to possible death. The model inputs include the number of cohort members in each CHD/CARP history state by age and gender at baseline (2001) and the values of all event risks for each age, gender and history state for each year of simulation (2001 to 2010). Events are simulated for each cohort member according to the event risks and this is continued until the cohort member dies, reaches the upper age cutoff (80 years), or the end of the simulation period (2010) whichever comes first. In the context of forecasting, population event risks for past years (1998 to 2000 in our case) are calculated and used to project event risks for future years. A total of 100 replicate simulations of the cohort are performed and the average event number (eg CARPS) over these 100 cohort simulation are used as the results of the simulation Full details of the model and a demonstration of its use for forecasting (from 1995 to 1999) have been described and discussed previously [17,18]. A schematic diagram of the Markov model is shown in Figure 1.

## Results

**Figure 2:**
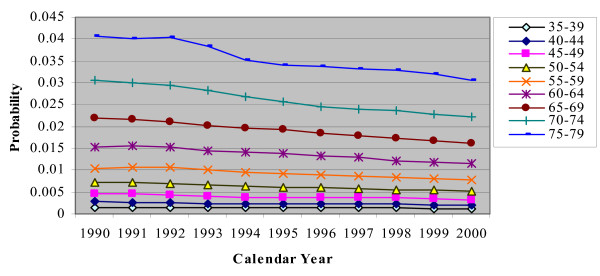
**Plot for Pr(CHD|history of no CHD) by calendar year, for males with different age groups**.

**Figure 3:**
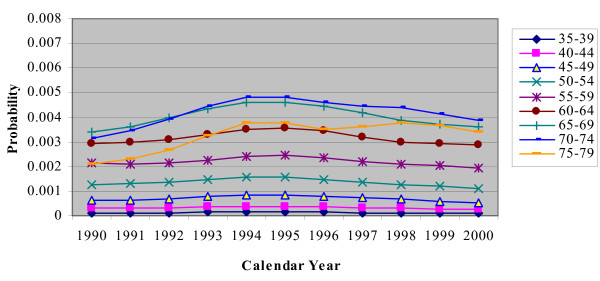
**Plot for Pr(a CABG|history of no CHD) by calendar year, for males with different age groups**.

**Figure 4:**
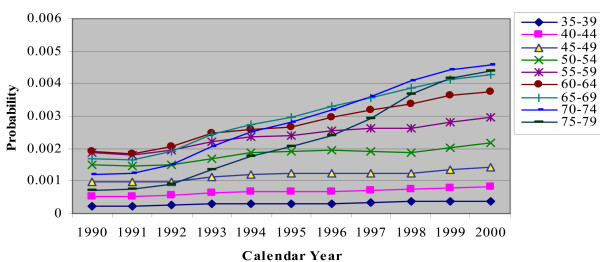
**Plot for Pr(a PCI|history of no CHD) by calendar year, for males with different age groups**.

**Figure 5:**
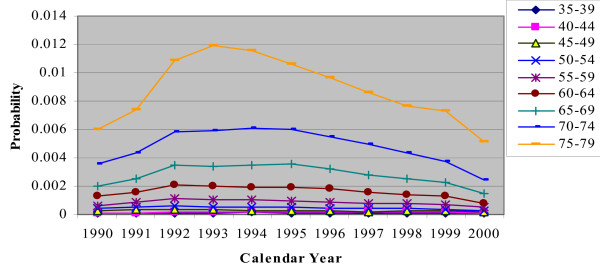
**Plot for Pr(CHD death|no CHD and no history of CHD) by calendar year, for males with different age groups**.

**Figure 6:**
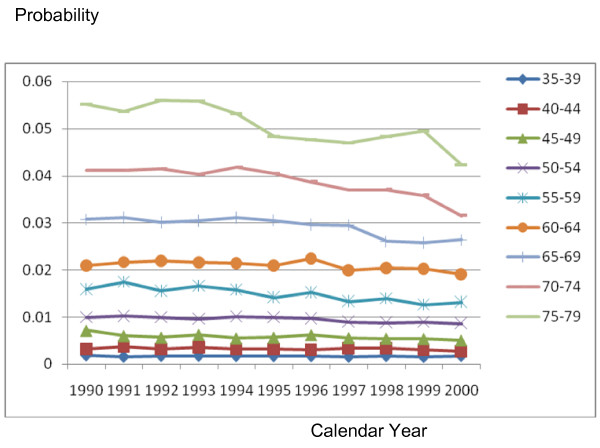
**Plot for incidence of CHD by calendar year, for males with different age groups**.

In the CHD/CARP model, incident CHD events are represented by the occurrence of a CHD admission (with or without a CARP) and CHD death (without a prior hospitalization) for people who have no history of CHD (ie no previous CHD admission or CARP). Figures 2, 3, 4, 5 and 6 show the estimated event probabilities relating to incident CHD events for the years from 1990 to 2000 by age for males (similar trends are observed for females). These show that in the period from 1995 to 2000 all the components of incident CHD have been declining except for (first-ever) hospital admissions in which a PCI is performed and the net effect is that total CHD incidence (Figure 6) has been declining. Also, these Figures show that the dominant (largest) component of CHD incidence is the risk of a CHD admission (without a CARP) for those with no history of CHD. For the older age groups, this risk declined very slowly during 1995-99 and at a rate lower than that of the period 1990-94. Thus, the rate of decline slowed down during the second half of the period 1990-99.

To investigate the impact of changing CHD incidence on CARPs requirements we have simulated the numbers of CABGs and PCIs for the 2001 WA population cohort over the period 2001 to 2010. A number of simulations are conducted. In all simulations the model uses event risks for 2001 to 2010 that are based on extrapolating the estimated linear trend in event risks over the years 1998 to 2000 into the period 2001 to 2010 but with certain CHD incident event risks modified. The modifications of event risks are described as follows and attempt to capture a range of possible CHD event risks relating to leveling out of some declining CHD incident event risks and even an increase in some CHD incidence components.

Base scenario: The linear trends observed over 1998 to 2000 in all event risks (including CHD incident events) are assumed to continue into 2001 to 2010.

Incidence (1) scenario: The Pr (CHD |no history of CHD) levels off while the trends in other event risks continue.

Incidence (2) scenario: The Pr(CHD|no history of CHD) is increased by 10% while the trends in other risks continue.

Incidence (3) scenario: The Pr (CHD| no history of CHD) is increased by 20% while the trends in other risks continue.

Incidence (4) scenario: The Pr(CHD|no history of CHD) is increased by 20%, the Pr(PCI| no history of CHD) is increased by 5%, and Pr (CABG |no history of CHD) and Pr(CHD death|no history of CHD) both level off, while the trends in other event risks continue.

Table 1 shows that the total simulated numbers of CABGs and PCIs increase by only 0.96% and decrease by only 1.58% respectively under Incidence(1) scenario compared to the Base scenario. This is because the risk of CHD for those with no history of CHD declined very slowly over the period 1998-2000, and thus when these were modified to level off and the remaining risks were linearly extrapolated into 2001-2010, the simulated numbers of CABGs and PCIs did not change much from those which were simulated under the Base scenario. This table further shows that under Incidence(2) and Incidence(3) scenarios the simulated numbers of CABGs increase by only 2.20% and 3.39% respectively as compared to the Base scenario. The corresponding increases for PCIs are 4.55% and 5.3% respectively. The change in simulated number of CABGs under Incidence(4) scenario compared to the Base scenario is more dramatic as this number is 25.16% greater than that obtained by the Base scenario. The corresponding increase for PCIs is 18.57%.

**Table 1:** Comparison between projected numbers of CARPs (2001-2010) based on linear, incidence(1), incidence(2), incidence(3) and incidence(4) scenarios

Total event	Base scenario	Modified(1) scenario	Modified(2) Scenario	Modified(3) Scenario	Modified(4) Scenario
CABG	10084	10181	10306	10426	12621
% change from linear extrapolation	---	0.96	2.20	3.39	25.16
PCI	26757	26333	27975	28174	30483
% change from linear extrapolation		-1.58	4.55	5.30	13.93

**Figure 7:**
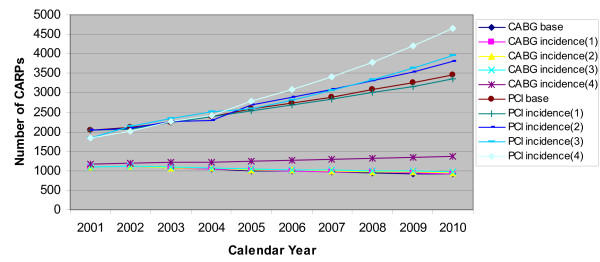
**Comparison between projected numbers of CABGs and PCIs by base, incidence(1), incidence(2), incidence(3) and incidence(4) scenarios over the period 2001-2010**.

Figure 7 shows the total projected numbers of CABGs and PCIs obtained under the various simulation scenarios by single year over 2001-2010. The differences between the simulated numbers of CABGs and PCIs obtained under Incidence(1) and Base scenarios are very small for every calendar year. Similarly, the respective differences between the simulated numbers of CABGs and PCIs obtained under Incidence(2) and Incidence(3) scenarios in comparison to Base scenario are small. However, the differences between the simulated numbers of CABGs and PCIs obtained under Incidence(4) and Base scenarios, are larger and generally increase with calendar year.

## Discussion and conclusions

In this study, we have used a previously validated CHD/CARP Markov simulation model for evaluating the effect of changing CHD incidence trends on requirements of CARPs. CHD incidence has been declining for some time but this trend might level out or even reverse due to the obesity epidemic. Recent data for obesity in WA indicate that mean BMI has increased at the rate of 0.175 kg/m^2 ^per year over the period 2002-2006 [22] which is faster than the rate of increase in mean BMI over 1983-1999 as reported by Hobbs et al [15]. These data support the underlying assumption for the development of our incidence scenarios. However, other risk factors still continue to improve in WA [22]. For example, mean levels of cholesterol actually declined over the same period, possibly because of a compensating increase in personal awareness of cholesterol levels and increased use of cholesterol lowering treatment. The net effect on CHD incidence is unclear but it is still worth exploring the range of possible effects on CARP requirements.

There are a number of factors, other than population risk factor levels, which could also influence trends for CHD incidence in the population based on hospital admission data. For example, a recent study in WA demonstrated that while the long-standing decline in hospital admission rates for MI appeared to level off after 1997 it is likely to have been an artefact related to more sensitive and specific biomarkers for myocardial damage (troponins) [23]. It should also be noted that "pre-emptive" CARPs performed in persons with sub-acute CHD such as angina pectoris could also have contributed to the downward trend in incident acute CHD without CARPs such as MI or unstable angina. CARP rates may also have been affected by broadening of clinical indications for coronary revascularization. For example, immediate (Primary) PCI is now the preferred method of coronary artery revascularization in acute coronary syndromes (MI or unstable angina) in place of thrombolytic (medical) treatment. There would thus be an apparent decrease in incident CHD without CARP and a reciprocal increase in CARP with CHD.

The requirements for CARPs may also be influenced by the changing trends in diabetes prevalence. In WA, the prevalence of diabetes has been increasing. However, one of the principal drivers for this change could very well be the obesity epidemic in the population. Thus, this again reiterates the indirect effect the obesity epidemic may have on the future requirements of CARPs.

In our study, we have developed four incidence scenarios in such a way that they represented a gradual deterioration in the favourable CHD incidence trends and assumed that other risks continued in their current trend pattern. The strategy adopted for developing the scenarios was that the most dominant component of CHD incidence was first gradually altered and finally the remaining components were altered. The results showed that if the most dominant component of CHD incidence, that is, the risk of CHD admission for those with no history of CHD levelled off and the trends in all other transition probabilities continued, then the projected numbers of CABGs and PCIs for 2001-2010 were insensitive to these changes. Even in the unlikely scenario of increasing this event probability by 20% did not alter the results much. However, when the other components of CHD incidence, namely risk of CABG for those with no history of CHD, risk of PCI for those with no history of CHD and risk of CHD death for those with no history of CHD, were projected to increase from the baseline period of 1998-2000 to 2001-2010, the requirements of CARPs increased substantially. Thus, this scenario analysis suggests that if there is increased CHD incidence involving an increased likelihood of a CARP in a person's first ever CHD hospital admission then there may be a substantial increase in the requirements of CARPs and hence a substantial increase in costs for managing CHD. However, if the increase involves more PCIs rather than CABGs then the increased cost burden may be less given the relative cost of the two procedures [24].

A limitation of this study is that it was initiated in 2004 when the complete collection of state-wide data was available up to 2000. Hence the model based estimates and trends applied to the period 1998 to 2000 and these were used to project the number of PCI and CABG in Western Australia over the period of 2001-2010. Whilst it is now already 2009 the sensitivity of CARP forecasts to various scenarios relating to changing CHD incidence are still broadly applicable and highlight the need to not only monitor the changing CHD incidence due to the obesity epidemic and rising prevalence of diabetes but also the need to examine the changing nature of initial presentation of CHD, that is a first-ever CHD hospital admission with or without a CARP.

A limitation of our study for investigating the effect of changes in risk factor trends on CARP requirements is that it does not directly incorporate population risk factor levels. Theoretically, it is possible to directly incorporate population risk factor data into the CHD/CARP model. Risk factor prevalence data from population surveys could be used to divide the population without history of CHD into risk factor strata and Framingham-type equations for projecting CHD incidence rather than the risk estimates for incident CHD used in the present study. In other words, we could replace the incidence risks by a separate incidence submodel similar to what the CHD Policy Model [25] does. However, to know the relative importance of CHD risk factors at the population level and to record changes in these factors over time requires person-based information about long-term trends in these factors and these data need to be linked longitudinally to relevant outcomes in the same individuals. Such data are not available at the population level in WA and in most populations.

In this study, we have presented only one particular use of the WA CHD/CARP Markov simulation model. As discussed earlier this model has already been used to explore scenarios evaluating the effect of advances in PCI on future requirements of CARPs [21]. However, a number of other applications remain to be explored. These include but are not limited to determining the effect on requirements of CARPs of advances in medical treatments related to CHD and changes in health policy related to treating CHD patients. Examples of determining effects of advances in medical treatments include increasing use of prescribed aspirin, lipid lowering drugs, statins and ACE inhibitors. Examples of examining effects on requirements of CARPs due to changes in health policy include change in health policy (by the Australian government) regarding subsidising statins by only providing subsidy to people at higher risk of CHD.

## Competing interests

The authors declare that they have no competing interests.

## Authors' contributions

HRM did all the modeling, programming and analyses as a doctoral candidate under the supervision of MK and MH. HRM prepared the first drafts of manuscripts from this study and MK and MH reviewed, revised and approved the manuscripts. Authors MK and MH conceived the modeling framework, and secured access to all the population level data. All authors read and approved the final manuscript.

## Pre-publication history

The pre-publication history for this paper can be accessed here:

http://www.biomedcentral.com/1471-2261/10/2/prepub

## References

[B1] Beaglehole R Stewart AW Jackson R Dobson AJ McElduff P D'Este K Declining rates of coronary heart disease in New Zealand and Australia, 1983-1993 American Journal of Epidemiology 1997 145 8 707 13 9125997 10.1093/aje/145.8.707

[B2] AIHW (Australian Institute of Health and Welfare) Mathur S Epidemic of coronary heart disease and its treatment in Australia. Cardiovascular Disease Series No. 20. AIHW Cat. No. CVD 21 2002 Canberra: Australian Institute of Health and Welfare

[B3] Kuulasmaa K Tunstall-Pedoe H Dobson A Estimation of contribution of changes in classic risk factors to trends in coronary-event rates across the WHO MONICA Project populations The Lancet 2000 355 675 687 10.1016/S0140-6736(99)11180-2 10703799

[B4] Booth ML Chey T Wake M Change in the prevalence of overweight and obesity among young Australians, 1996-1997 American Journal of Clinical Nutrition 2003 77 29 36 12499319 10.1093/ajcn/77.1.29

[B5] AIHW (Australian Institute of Health and Welfare) Australia's Health 2006. AIHW Cat. No. AUS 73 2006 Canberra: Australian Institute of Health and Welfare

[B6] Barr ELM Marliano DJ Zimmet PZ AusDiab 2005: the Australian diabetes, obesity and lifestyle study 2006 International Diabetes Institute, Melbourne, Australia

[B7] Krieger DR Landsberg L Mechanisms in obesity-related hypertension: role of insulin and catecholamines, American Journal of Hypertension 1998 1 84 90 10.1093/ajh/1.1.84 3285861

[B8] Denke MA Sempos CT Grundy SM Excess body weight: an unrecognized contributor to dyslipidemia in white American women Journal of Internal Medicine 1994 154 401 410 10.1001/archinte.154.4.401 8117172

[B9] Denke MA Sempos CT Grundy SM Excess body weight: an unrecognized contributor to high blood cholesterol levels in white men Archives of Internal Medicine 1994 153 1093 103 10.1001/archinte.153.9.1093 8481076

[B10] NHLBI Obesity Education Initiative Expert Panel Clinical Guidelines: Identification, Evaluation, and Treatment of Overweight and Obesity in Adults: The Evidence Report 1998 Bethesda, MD: National Institutes of Health, National Heart, Lung, and Blood Institute

[B11] Keys A Overweight, obesity, coronary heart disease, and mortality Nutrition Review 1994 153 1093 103 10.1111/j.1753-4887.1980.tb05967.x 7432707

[B12] Larsson B Bjorntorp P Tibblin G The health consequences of moderate obesity International Journal of Obesity 1981 5 97 116 6971814

[B13] Simopoulos AP van Itallie TB Body weight, health, and longevity Annals of Internal Medicine 1984 100 285 95 6362514 10.7326/0003-4819-100-2-285

[B14] Barrett-Connor EL Obesity, atherosclerosis, and coronary heart disease Annals of Internal Medicine 1985 103 1010 19 3904565 10.7326/0003-4819-103-6-1010

[B15] Hubert HB The importance of obesity in the development of coronary risk factors and disease: the epidemiologic evidence Annual Review of Public Health 1986 7 493 502 10.1146/annurev.pu.07.050186.002425 3718653

[B16] Hobbs MST Knuiman MW Briffa T Ngo H Jamrozik K Plasma cholesterol levels continue to decline despite the rising prevalence of obesity: population trends in Perth, Western Australia, 1980-1999 Eur J Cardiovasc Prev Rehabil 2008 15 319 324 10.1097/HJR.0b013e3282f3c76b 18525387

[B17] Catenacci VA Hill JO Wyatt HR The Obesity Epidemic Clinics Chest Medicine 2009 30 3 415 444 10.1016/j.ccm.2009.05.001 19700042

[B18] Aitken RJ Allman-Farinelli MA King LA Current and future costs of cancer, heart disease and stroke attributable to obesity in Australia: a comparison of two birth cohorts Asia Pacific Journal of Clinical Nutrition 2009 18 1 63 70 19329397

[B19] Mannan HR Knuiman M Hobbs M A Markov simulation model for analysing and forecasting the number of coronary artery revascularization procedures in Western Australia Annals of Epidemiology 2007 17 12 964 975 10.1016/j.annepidem.2007.05.016 18022536

[B20] Mannan H Knuiman M Hobbs M Adapting a Markov Monte Carlo simulation model for forecasting the number of Coronary Artery Revascularisation Procedures in an era of rapidly changing technology and policy BMC Medical Informatics & Decision making 2008 8 27 10.1186/1472-6947-8-27 PMC2443119 18578858

[B21] Mannan H Knuiman M Exploring the likely effect of the introduction of drug eluting stents on requirements for coronary artery revascularisation procedures in Western Australia: A use of the CHD/CARP Markov simulation model The Open Epidemiology Journal 2009 2 42 46 10.2174/1874297100902010034

[B22] Wood N Daly A Heath and wellbeing of adults in Western Australia 2006, Trends over time for selected chronic conditions and risk factors 2007 Department of Health, Western Australia

[B23] San Filippo FM Hobbs MST Knuiman MW Hung J Impact of New biomarkers of Myocardial Damage on Trends in Myocardial Infarction Hospital Admission Rates from Population-based Administrative Data American Journal of Epidemiology 2008 168 2 225 233 10.1093/aje/kwn107 18468989

[B24] Hailey D Banta HD, Gelband H, Battista RN, Jonsson E Health care technology in Australia Health care technology and its assessment in eight countries 1994 Office of Technology Assessment. United States Congress

[B25] Weinstein MC Coxson PG Williams LW Pass TM Stason WB Goldman L Forecasting coronary heart disease incidence, mortality, and cost: the Coronary Heart Disease Policy Model American Journal of Public Health 1987 77 11 1417 26 10.2105/AJPH.77.11.1417 3661794 PMC1647098

